# Prognostic value of Killip classification in terms of health-related quality of life

**DOI:** 10.1186/cc10789

**Published:** 2012-03-20

**Authors:** A Ioannidis, D Tsounis, A Pechlevanis, M Paraskelidou

**Affiliations:** 1 HOU, Kalamaria, Greece; 2 GHT 'Agios Pavlos', Kalamaria, Greece

## Introduction

The aim of the study was to evaluate the prognostic value of Killip classification in terms of health-related quality of life (HRQoL).

## Methods

The sample consisted of 112 patients treated for myocardial infarction (MI), as onset manifestation of coronary artery disease (CAD), during 2008/09 in a prefectural hospital in northern Greece. At 1-year follow-up visit, HRQoL was measured using a generic and a disease-specific instrument. The 15D consists of a visual analogue scale (VAS) and a total score. The scoring algorithm of the MacNew generates a global score, and three separate domains scores: emotional, physical and social.

## Results

Patients were grouped into the four Killip classes according to the degree of pulmonary congestion at admission (Table 1). Mean HRQoL for each group differed in the expected manner: the higher the class, the lower the HRQoL. Statistical significant differences were observed in VAS of the 15D and the emotional and social domain scores of the MacNew. Accordingly, the majority of patients with no signs of pulmonary congestion at admission were classified in NYHA functional class I at 1-year follow-up visit. No difference was observed in the type of MI, although patients in higher Killip classes had more affected arteries and were treated more often with CABG than PCI. Additionally, Killip class I patients were favored in a number of parameters including age, systolic blood pressure, heart rate, BMI, NT-proBNP level and LVEF.

**Table 1:** Patient characteristics according to Killip classification

	Class I	Class II	Class III	Class IV	*P *value
*n *(%)	52 (46.4)	40 (35.7)	14 (12.5)	6 (5.4)	NA
HRQoL (mean)					
15D					
VAS	78.9	76.9	72.9	70.8	**0.025**
Total	0.844	0.842	0.823	0.803	0.478
MacNew					
Emotional	5.60	5.61	5.38	5.05	**0.030**
Physical	5.49	5.26	5.25	5.05	0.144
Social	5.72	5.47	5.30	5.14	**0.014**
Global	5.52	5.44	5.27	4.98	0.056
Age (years)	61.8	66.0	70.2	61.2	**0.013**
Gender (*n*, %)					
Male (85)	40 (47.1)	31 (36.5)	12 (14.1)	2 (2.4)	0.080
Female (27)	12 (44.4)	9 (33.3)	2 (7.4)	4 (14.8)	
ΒΜΙ	28.7	29.1	30.0	34.8	**0.001**
Systolic BP	127.5	122.5	115.7	140.0	**0.000**
Diastolic BP	75.8	73.6	70.7	76.7	0.173
Heart rate	64.9	68.7	72.3	71.7	**0.030**
CRP	3.6	4.9	6.0	3.8	0.087
NT-proBNP	741	1645	2193	1674	**0.000**
LVEF	61.9	55.2	49.0	45.0	**0.000**
MI type (*n*, %)					
STEMI (48)	24 (50.0)	18 (37.5)	4 (8.3)	2 (4.2)	0.638
NSTEMI (64)	28 (43.8)	22 (55.0)	10 (15.6)	4 (6.3)	
Affected arteries (*n*, %)					
1 (38)	19 (50.0)	15 (39.5)	4 (10.5)	0 (0.0)	**0.005**
2 (30)	17 (56.7)	9 (30.0)	4 (13.3)	0 (0.0)	
3 (44)	16 (36.4)	16 (36.4)	6 (13.6)	6 (13.6)	
Revascularization technique (*n*, %)					
None (14)	7 (50.0)	3 (21.4)	4 (28.6)	0 (0.0)	**0.002**
PCI (63)	35 (55.6)	22 (34.9)	6 (9.5)	0 (0.0)	
CABG (35)	10 (28.6)	15 (42.9)	4 (11.4)	6 (17.1)	
ΝΥΗΑ					
Ι (73)	42 (57.5)	21 (28.8)	8 (11.0)	2 (2.7)	**0.013**
ΙΙ (37)	8 (21.6)	19 (51.4)	6 (16.2)	4 (10.8)	
ΙΙΙ (2)	2 (100.0)	0 (0.0)	0 (0.0)	0 (0.0)	
Rho	0.28				**0.001**

## Conclusion

Patients with MI as an onset manifestation of CAD present with varied degrees of pulmonary congestion. The prognostic value of the Killip classification is highlighted in terms of the extent of CAD (number of affected vessels and revascularization technique), of NYHA class and, last but not least, of HRQoL.

